# A Molecular Modeling Study on the Propagation in Free Radical Chain Oxidation of (B)PEI

**DOI:** 10.3390/nano15040313

**Published:** 2025-02-18

**Authors:** Wim Buijs

**Affiliations:** Independent Researcher, Lochlehn 237, 6105 Leutasch, Austria; wbuijsm@gmail.com

**Keywords:** density functional theory, Free Radical Chain Autoxidation, Basic Autoxidation Scheme

## Abstract

Air oxidation of PEI is a Free Radical Chain Autoxidation process, described as a process following the Basic Autoxidation Scheme with Initiation, Propagation and Termination as discriminating steps. Molecular Modeling was able to identify the most important propagation reactions. HO_2_(d) is the most likely candidate as the main oxidation chain carrying radical. α-H-abstraction from PEI α-amino hydroperoxides by HO_2_(d), leading to amide PEI repeat units and eventually to HO_2_(d) again, is the first step in Propagation. Apart from well-know propagation reactions, the reaction of PEI α-amino CH(d) radicals with H_2_O_2_ is of major importance, too, with an estimated contribution of ~50% to Propagation. Furthermore, it provides an explanation for the formation of NH_3_ and various imine PEI repeat units. PEI α-amino alkoxy radicals might contribute to some extent to Propagation and can lead to chain breaks in PEI and the formation of CO_2_. Amide and imine PEI repeat units contribute to ~90% of the fully oxidized PEI.

## 1. Introduction

### 1.1. CO_2_ Capture and PEI

CO_2_ capture is an important emerging technology to mitigate climate change [1,2]. Amine resins are intensively investigated as potential materials for CO_2_ capture both from air and large point emission sources, like power plants and energy-intensive industries [3,4,5,6,7,8,9]. They show good reversible CO_2_ uptake and release; however, their long-term oxidation stability might be a drawback for practical application [10,11,12,13,14,15,16]. This is particularly true for Direct Air Capture (DAC) of CO_2_, which inherently deals with widely varying conditions with respect to temperature, humidity, and contaminations, even within one day.

Among these resins, PEI (Poly Ethylene Imine) is well known as a potentially suitable material for CO_2_ capture [5]. PEI is a collective name for a series of polymers with different molecular weights, which can be divided into two main categories: LPEI (Linear Poly Ethylene Imine) and BPEI (Branched Poly Ethylene Imine). These differences do not only affect their structures—LPEI shows (a) crystal structure(s) and is (partly) solid until 50–110 °C [17,18,19,20,21,22], while BPEI is usually a viscous liquid [23,24]—but also their chemical (catalytic) reactivity not only in CO_2_ capturing reactions but also in oxidative degradation. Parts of the oxidative degradation of organic polymers are not well understood due to the enormous variety of oxidation products and varying reaction rates. The simple repetitive structure of PEI could allow identification of all major oxidation products and reaction mechanisms. The identification of these products and reaction mechanisms transcends the importance of PEI and might contribute to the understanding of oxidative degradation of organic polymers in general.

### 1.2. Experimental Results on the Oxidation of PEI from Literature

Air oxidation of organic polymers proceeds via the so-called Free Radical Chain Autoxidation (FRCA) or Basic Autoxidation Scheme (BAS) [25,26]. FRCA is not limited to the oxidative degradation of polymers but is also observed in the air oxidation of bulk chemicals such as cyclohexane [27,28]. Nezam et al. reported on the chemical kinetics of the oxidation of poly-ethylenimine in CO_2_ sorbents [10]. In this study, PEI-800 was used. This is a BPEI oligomer with an average molecular weight of 800 g/mol according to LS and 600 g/mol according to GPC [23]. This corresponds to an average chain length of 18 (LS) ethylenimine units with NH_2_-end-groups. They carried out their oxidation study under dry conditions. The extent of oxidation was measured using two techniques: (a) loss of amine efficiency to capture CO_2_, and (b) Differential Scanning Calorimetry (DSC). In addition, Thermogravimetric Analysis (TGA) and elemental analysis were conducted. They found that loss of amine efficiency is always faster than the heat production measured by DSC. Total oxidation was obtained in approximately 250–1000 min, with temperatures ranging from 150 °C to 125 °C. Still, 82–87% of the mass of BPEI was retained at 5–30% of O_2_. The normalized C/N ratio increases from 1.0 to 1.2 from the start to total oxidation, while the normalized H/(C+N) ratio decreases from 1.0 to 0.7. This accounts for a loss of ~20% of the amine groups, converted either to NH_3_ or other volatile N-containing products.

The activation barrier was measured as a function of the extent of oxidation. After a short induction period, an average activation barrier of ~105 kJ/mol ± 10 kJ/mol was obtained in the oxidation range from 10 to 70%. Beyond 70%, oxidation the activation barrier drops rapidly to <30 kJ/mol at almost total oxidation.

The reaction order in O_2_ was found to be between 0.5 (125 °C) and 0.7 (150 °C) up to ~50% extent of oxidation, whereafter, it drops to almost 0.

In a follow-up study, Racicot et al. described the formation of volatile products from the autoxidation of Pei-800 [11]. It was found that NH_3_, H_2_O, and CO_2_ were produced in a rather constant ratio during the autoxidation process. At the end of the autoxidation process, the ratio of NH_3_/N-BPEI was ~0.21, that of H_2_O/H-PEI was ~0.10, and that of CO_2_/C-PEI was ~0.02.

Apart from experimental results, some computational results were presented, too. An attempt to account for the formation of NH_3_, using Tri-Ethylene-Tetra-Amine (TETA) as a small model for BPEI and applying a metadynamics approach, yielded a ΔG_act_ = 282 kJ/mol for the direct elimination of NH_3_ from TETA, while a process starting from the TETA β-radical yielded NH_2_(d) and the corresponding N-vinyl-Di-Ethylene-Tri-Amine (N-vinyl-DETA) with a ΔG_act_ ~90 kJ/mol.

Ahmadalinezhad and Sayari [13] studied oxidative degradation of LPEI and BPEI impregnated on mesoporous silica at various temperatures. They applied a variety of NMR techniques to identify structural elements in the oxidated PEI. They found that both oxidized LPEI and BPEI contained RNHCH_2_-C=O-NHR* and RNH-CH_2_-CH=NR* units, but RNH-C=O-CH=NR* units were observed in oxidized BPEI only.

Min et al. observed in their work on the oxidation stability of BPEI [14,24] a sharp increase in an IR adsorption at 1670 cm^−1^, which was related to the formation of C=O and/or C=N bonds and a decrease in IR adsorptions between 2800 and 3000 cm^−1^, which was related to the disappearance of C-H bonds in oxidized BPEI. The C=O and/or C=N bonds were attributed to the formation of amides and imines in line with the findings of Ahmadalinezhad and Sayari [13].

### 1.3. Computational Results from Literature

The oxidation of cyclohexane is an important industrial process with an annual production of ~10^6^ tons/year. This reaction proceeds via an FRCA mechanism. In the propagation step, a cyclohexyl radical (Cy (d)) reacts with O_2_(t) to yield the cyclohexylperoxy radical (CyOO(d). In the next step, the cyclohexylperoxy radical abstracts an H-atom from cyclohexane (CyH) to yield cyclohexyl hydroperoxide (CyOOH) and a new cyclohexyl radical:

Cy(d) + O_2_(t)     → CyO_2_(d) (1)

CyO_2_(d) + CyH    → CyO_2_H + Cy(d) (2)

In the classical description of FRCA, decomposition of CyO_2_H into CyO•(d) and HO(d) is thought to contribute to *propagation*, as HO(d) will abstract an H-atom from CyH to yield Cy(d) and H_2_O. Product formation to cyclohexanone (Cy=O) and cyclohexanol (CyOH) is considered a *termination step* from the reaction of two CyO_2_(d) radicals:

2 CyO_2_(d)    → Cy=O + CyOH + O_2_(t) (3)

Hermans et al. [27] experimentally observed the rapid formation of CyO_2_H, Cy=O, and CyOH as a function of conversion at 145 °C and concluded that product formation to Cy=O and CyOH must originate from fast *propagation* steps. Using DFT calculations, they found that the activation barriers for α-H-atom abstraction from CyO_2_H and CyOH by CyO_2_(d) are ~24 and 22 kJ/mol, respectively, lower than from CyH, and the corresponding reaction rate was 20–80 and 5–20 times faster. Thus, α-H-atom abstraction starting from CyO_2_H and CyO_2_(d) is an important channel for propagation. The formal initial product of that reaction, α-Cy(OOH)(d), decomposes directly into Cy=O and HO(d), which contributes to the propagation also.

CyO_2_H + CyO_2_(d)   → CyO_2_H + α-Cy(OOH)(d) α-Cy(OOH)(d)  → Cy=O + HO(d)(4)

The formation of CyOH was explained by another *propagation* step instead of a *termination* step:

CyO_2_(d) + CyH    → CyO_2_H + Cy(d) (5)

CyO_2_H + Cy(d)     → CyOH + HO(d) (6)

As Reaction (6) would be too slow in the bulk liquid phase, the authors had to assume that this reaction takes place in a so-called “Franck–Rabinowitch” solvent cage [29]. At higher conversion, a major role in the propagation process is taken by HO(d), which leads to the formation of CyO(d). The CyO(d) radical yields ~60–70% CyOH by H-atom abstraction and 40–30% ring-opening b-C-C cleavage products. Thus, most of the byproducts in cyclohexane oxidation do not originate from cyclohexanone but from CyO(d).

In a continuation of the former study, Hermans et al. [28] addressed the experimental finding that cyclohexanone, amongst other ketones, clearly enhances the cyclohexane oxidation rate in the early stage of the oxidation. They proposed an alternative reaction of CyO_2_H with Cy=O and CyH:

CyO_2_H + Cy=O    → CyO•(d) + H_2_O + Cy=O α-CH(d) and (7)

CyO_2_H + CyH     → CyO•(d) + H_2_O + Cy(d) (8)

This type of reaction requires a lower activation barrier of homolytic dissociation of CyO_2_H into CyO(d) + HO(d), which is about 170 kJ/mol. The authors were able to locate a transition state with an activation barrier of ~100 kJ/mol. This reaction can be considered as a modification of the old mechanism wherein homolytic dissociation of ROOH into RO(d) and HO(d) is a crucial step in the propagation [25,30].

### 1.4. Molecular Modeling and Chemical Engineering Modeling Results for Air Oxidation of Toluene

Air oxidation of toluene is a FRCA process like air oxidation of cyclohexane, and Hermans et al. [31] have also published a study on the air oxidation of toluene. An important finding is that HO_2_(d) gradually replaces the role of the benzylperoxy radical as chain-carrying radical in propagation. In 2005, a study appeared by Hoorn et al. [32] on the incorporation of mass transfer phenomena in the air oxidation of toluene, which is often neglected in kinetic studies. It was concluded that, under industrial conditions (T = 140–160 °C, P = 4–7 bar), the overall toluene oxidation rate is slow in comparison to the mass transfer rate of oxygen.

## 2. Materials and Methods

Molecular Modeling calculations were conducted with Spartan ’20 and ’24 of Wavefunction [33]. This commercial package contains many methods, ranging from Molecular Mechanics, semi-empirical, and Hartree-Fock to Density Functional Quantum Mechanical calculations, applying codes well described in the literature. In addition, specific tasks are specified to ease the setup of calculations. For this article, the tasks Conformer Distribution, Equilibrium Geometry, Energy Profile, and Transition State Geometry were used frequently. Guesses for Transition State Geometries were quite often obtained via Energy Profiles, wherein the distance between the presumed reaction centers stepwise was lowered until they were within chemical bonding reach. Finally, a wide variety of properties can be calculated, ranging from thermodynamic data to various spectra (NMR, UV, IR, Raman), which allows for the comparison with experimental data. Both thermodynamic data and IR spectra were frequently used.

A crucial step in Molecular Modeling is finding good starting structures for the calculations. While this is easy for small and rigid molecules, it turns out to be a tedious task for larger flexible molecules, like the oligomers of PEI. Molecular Mechanics is often the only possibility due to the extremely large numbers of conformers, the reliability of the method with respect to structure and relative energy, and the apparent lack of experimental data on conformers and their distribution at equilibrium. The Merck Molecular Force Field (MMFF) forcefield [34] was used to obtain Conformer Distributions. To the best of my knowledge, this is the only forcefield wherein a comparison was made between computational results and scarce experimental data. An additional advantage of Molecular Mechanics is the explicit description of Van der Waals interactions.

The candidate structures of PEI oligomers obtained with MMFF were used as input for quantum chemical calculations (Equilibrium and Transition State Geometries) using density functional theory. Wherever possible, MMFF structures were used as a guide to create even smaller models while still keeping essential elements of the original larger structures, but for some transition state structures, the semi-empirical method PM3 was used as input for DFT calculations. This not only saves computational time but also reduces the absolute error in DFT calculations with respect to size and limited account of dispersive (Van der Waals) interactions. The latter is an intrinsic problem for most DFT codes because DFT calculations yield mean electron densities of molecular systems. Attempts to overcome this, for example, that of Grimme [35] by reparameterization of DFT codes, leading to DFT-D3 codes, are only partly successful and can cause other problems [36,37]. Therefore, B3LYP/6-31G* was applied because it belongs to the most applied and validated codes, and its merits and drawbacks are well known. The error in DFT calculations was further reduced by applying as much as possible the concept of isodesmic reactions [38], which led to the cancellation of errors in estimates for the reaction enthalpy and activation enthalpy. Transition states were characterized by their unique imaginary frequency or internal reaction coordinate (IRC) [39]. Reaction energies and activation barriers were estimated from the sum of B3LYP/6-31G* total energies and enthalpy corrections. Entropy corrections were not applied due to the large simplifications of the models used for DFT calculations.

## 3. Computational Results

### 3.1. Model Systems

**Figure 1:**
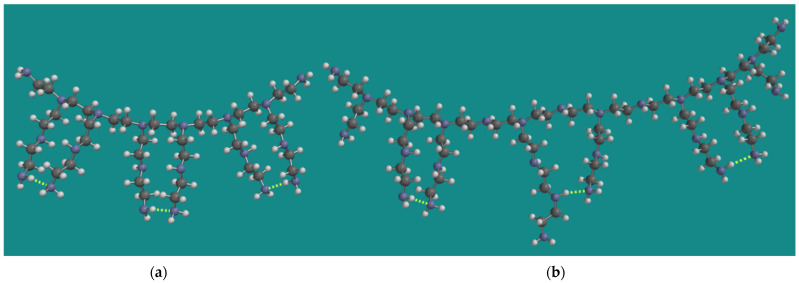
MMFF BPEI models for (**a**) PEI-800 Sigma-Aldrich PN 408719 [23] and (**b**) Epomin SP-012 [24]; display: ball and spoke, C: grey, N: blue, H: white, hydrogen bridges: yellow.

As described above, the experimental work of Nezam et al. used BPEI with an average molecular weight of 800 g/mol and a corresponding average chain length of 18 [10,23]. Min et al. used a BPEI with an average molecular weight of 1200 g/mol and a corresponding average chain length of 27 [14,24]. Figure 1 shows a model for both types of BPEI.

The models were constructed as described in detail previously by Buijs [15]. The molecular weight of the model for PEI-800 is 834 g/mol, corresponding to a chain length of 19. The amine composition is primary/secondary/tertiary = p/s/t = 8/6/6 or 40%/30%/30%. The molecular weight of the model for Epomin SP-012 is 1179 g/mol, corresponding to a chain length of 27. The amine composition is primary/secondary/tertiary = p/s/t = 10/10/8 or 36%/36%/28%, close to the analytical result of Min et al. [14]. An important aspect of the MMFF models for BPEI is their CO_2_ capacity, not only for material efficiency reasons but also to determine the amount of oxidation as described above. Under dry conditions, two amine groups are required to capture one CO_2_ molecule [40,41]. The maximum CO_2_ capacity of the model for PEI-800 is 3.6 mmol CO_2_/g PEI-800, in line with the experimental observation of Nezam et al. [10]. With respect to that property, the display of the model for Epomin SP-012 is a bit misleading, as it suggests that, in this case, a similar amount of CO_2_ can be captured, corresponding to 2.5 mmol CO_2_/g Epomin SP-012. However, the important characteristic of the model is the chain length, counted from the tertiary N-atoms. They should be at least two units long to allow two amines to come close enough to capture CO_2_. This could be two primary amines, but a combination of a secondary and a primary amine is also possible. Furthermore, the distance between the tertiary N-atoms can be one or two units. The total number of possible energetically favorable amine–amine interactions is, then, five. This leads to 4.2 mmol CO_2_/g Epomin SP-012, which is close to the value experimentally observed by Min et al. [14] of 4.0 mmol CO_2_/g Epomin SP-012. Of course, various permutations of this structure can be made with a similar outcome.

**Figure 2:**
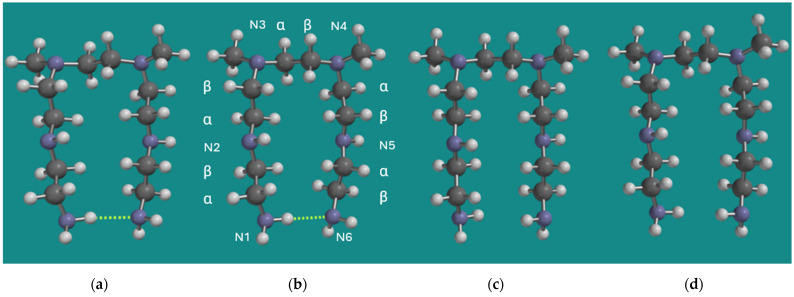
N,N-3,4-dimethyl N6 pentamer as a small BPEI model for PEI-800 Sigma-Aldrich PN 408719 [23] and Epomin SP-012 [24]; (**a**) H-bridged, MMFF, (**b**) H-bridged, B3LYP/6-31G*, (**c**) non-H-bridged, MMFF, (**d**) non-H-bridged, B3LYP/6-31G*. Display: ball and spoke, C: grey, N: blue, H: white, hydrogen bridges: yellow.

The MMFF structures of BPEI were used as input for quantum chemical calculations using B3LYP/6-31G* as described in Section 2. Materials and Methods. A common structural element in both models is the one where the tertiary N-atoms are separated by one unit only, and the chain lengths of the units on the tertiary N-atoms are two. Therefore, the earlier applied and smaller model N,N-3,4-dimethyl N6-pentamer of PEI was applied. As a control, a full Conformer Distribution on this smaller model was determined using MMFF. The total number of formal conformers for this small model is already >2.29*10^8^. An energy threshold for conformers ≤ 20 kJ/mol above the strain energy of the best conformer was added to ease the analysis of computational results. The best conformer (MMFF) is shown in Figure 2, together with its non-amine H-bridged counterpart, both as MMFF and B3LYP/6-31G* geometry-optimized structures. In addition, the numbering of the six N-atoms and the α, β numbering of the CH_2_ groups is provided under (b).

Visually, the structures (a) and (b) on one side and (c) and (d) on the other side are very similar. The largest difference is the length of the RNH_2_–NH_2_R H-bridge, which is 2.198 Å for the MMFF structure and 2.324 Å for the B3LYP/6-31G* structure. Furthermore, the energy difference between the H-bridged and the non-H-bridged structures is 9.1 kJ/mol for the MMFF structure and 9.9 kJ/mol for the B3LYP/6-31G* structure in both cases in favor of the H-bridged structures. So, even though DFT methods like B3LYP are known to underestimate dispersive interactions, there is no serious discrepancy between the results obtained with MMFF and B3LYP/6-31G* in this particular case, as relatively strong average electrostatic interactions are dominant.

### 3.2. Loss of Amine Efficiency

**Figure 3:**
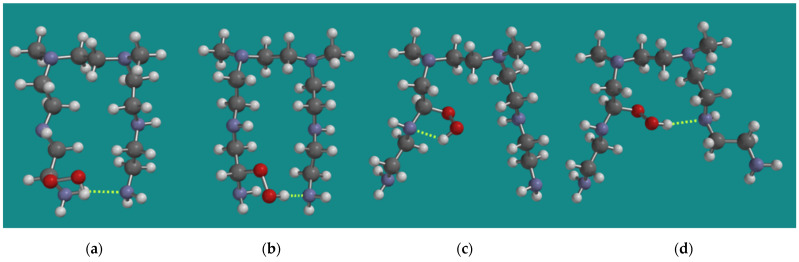
Various MMFF N,N-3,4-dimethyl N6 pentamer amino hydroperoxides; (**a**) N1-α-amino hydroperoxide NH-N H-bridged, (**b**) N1-α-amino hydroperoxide ROOH-N H-bridged, (**c**) N2-α-amino hydroperoxide intra-chain ROOH-N H-bridged, (**d**) N2-α-amino hydroperoxide inter-chain ROOH-N H-bridged. Display: ball and spoke, C: grey, N: blue, H: white, hydrogen bridges: yellow.

One of the observations of Nezam et al. [10] was that, during the progress of FRCA of BPEI, the loss of amine efficiency is always faster than the reaction heat production. An attractive explanation for that observation comes from the results from Molecular Modeling on the relative stability of the conformers of various α-amino hydroperoxides. Figure 3 shows various MMFF N,N-3,4-dimethyl N6 pentamer α-amino hydroperoxides. The formation of an α-amino hydroperoxide, in most cases, causes a substantial change in the conformation of the original N,N-3,4-dimethyl N6 pentamer, leading to geometries where the two amino groups, which are required to capture CO_2_, no longer are in each other vicinity. Though in the case of an α-amino hydroperoxide from a primary amine, an amine–amine H-bridge still seems possible, as can be seen in Figure 3a; the hydroperoxide–amine H-bridge shown in Figure 3b is 19.4 kJ/mol, which is more favorable than the amine–amine H-bridge shown in Figure 3a. In the case of an α-amino hydroperoxide from a secondary amine, amine–amine H-bridges are no longer possible, neither in (c) nor in (d). The α-amino hydroperoxide (c) is 34.0 kJ/mol higher in strain energy than (b), while α-amino hydroperoxide (d) is 61.3 kJ/mol higher in strain energy than (b). Thus, during the initial stage of the FRCA of BPEI, a loss of amine efficiency can already be expected.

### 3.3. FRCA of PEI: Propagation

#### 3.3.1. Decomposition of α-Amino Hydroperoxides by HO_2_(d) and HO(d)

Decomposition of the α-amino hydroperoxide by a radical is the first step in the propagation. Several options are available and will be discussed:

R_2_NCH(O_2_H)CH_2_NR_2_ + R_2_NCHO_2_(d)CH_2_NR_2_ → R_2_NC=OCH_2_NR_2_ + R_2_NCH(O_2_H)CH_2_NR_2_ + HO(d) (9)

R_2_NCH(O_2_H)CH_2_NR_2_ + HO_2_(d)     → R_2_NC=OCH_2_NR_2_ + H_2_O_2_ + HO(d) (10)

R_2_NCH(O_2_H)CH_2_NR_2_ + HO(d)     → R_2_NC=OCH_2_NR_2_ + H_2_O + HO(d) (11)

Reaction (9) is the analog of Reaction (8) discussed by Hermans et al. [28]. In principle, the reaction should be as plausible as in cyclohexane oxidation. However, the diffusion coefficient of the BPEI oligomer, PEI-800, is too low. By applying the classical relation of Wilke and Chang [42] and using the molecular volumes of the PEI-800 model and HO_2_(d) from their MMFF structures, it can be estimated that the diffusion coefficient of PEI-800 will be ~8* smaller than the diffusion coefficient of HO_2_(d). Thus, for BPEI, this reaction is not likely to occur. In addition, the same reaction inside an oligomer, between two chains in close vicinity of each other, also seems unplausible because of steric hindrance, as can be seen in Figure 3. Therefore, Reaction (10) seems much more likely, as HO_2_(d) is mobile, relatively stable, and can build up to some extent. Thus, the HO_2_(d) radical can diffuse to other PEI-800 oligomers and make a large contribution to the FRCA chain length. H_2_O_2_ itself might contribute to the propagation chain by reacting with HO(d) to yield H_2_O and HO_2_(d), although it decomposes in H_2_O and O_2_(t) too [43,44]. Reaction 11 could be considered the direct counterpart of Reaction (10) in the propagation. However, the HO(d) radical is extremely reactive and could easily abstract a variety of H-atoms available, including the H-atoms of H_2_O_2_ and HO_2_(d), yielding H_2_O and HO_2_(d) or O_2_(t) in almost barrier-free reactions.

Table 1 provides an overview of all propagation reactions investigated, starting from reactions of various hydroperoxides with either HO_2_(d) or HO(d), reactions starting from the N,N-3,4-dimethyl N6 pentamer with either N1-α-amino peroxy (d) or HO_2_•(d) and reactions from H_2_O_2_ with α-amino radicals of the N,N-3,4-dimethyl N6 pentamer. Reactions of α-amino radicals with O_2_(t) to yield α-amino hydroperoxyl radicals are barrier free and are not listed.

**Figure 4:**
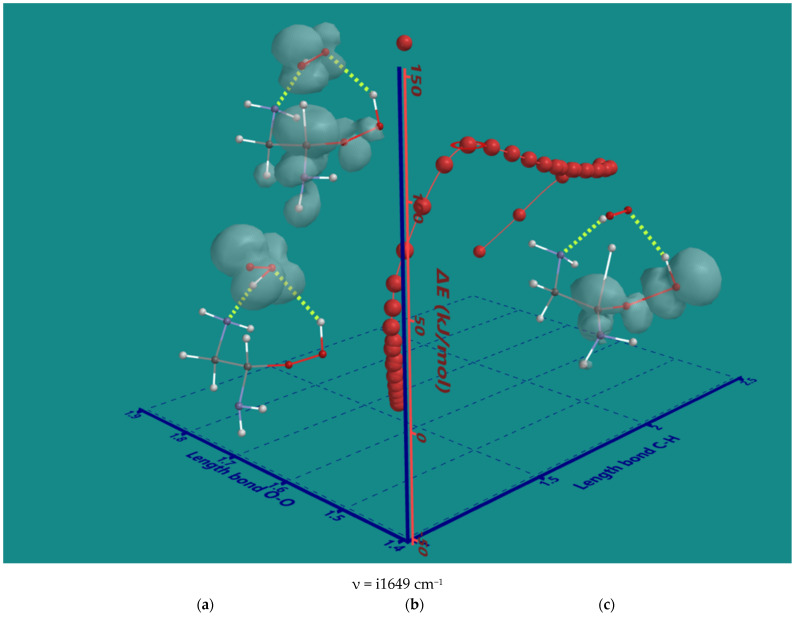
B3LYP/6-31G* IRC of the decomposition α-amino hydroperoxide of ethylene diamine with HO_2_(d); (**a**) starting complex, (**b**) transition state, (**c**) product complex. Display: ball and wire, C: grey, N: blue, H: white, hydrogen bridges: yellow; surface: spin density at isosurface = 0.002 e/au^3^. ΔE is ΔE-total energy. Bond lengths in Å.

The nature of the decomposition of the α-amino hydroperoxides with HO_2_(d) was investigated using α-amino hydroperoxide of ethylene diamine as the smallest possible system. An IRC plot of the reaction is shown in Figure 4, providing important details.

The original 2D IRC plot with ΔE-total energy vs. intrinsic reaction coordinate was converted into a 3D plot with ΔE-total energy vs. the α-C-H bond length and O-O bond length of the starting hydroperoxide, both essential for understanding the nature of the reaction. In the starting complex (a), the spin density is on HO_2_(d) only. Furthermore, HO_2_(d) is stabilized by two H-bridges, one with an amine and one with hydroperoxide. The H-bridges stay intact during the entire reaction. Firstly, HO_2_(d) moves to a more favorable position to abstract the α-H atom with no serious elongation of the C-H bond. Next, the C-H bond is elongated to 1.372 Å in the transition state (b). Its unique imaginary frequency of i1649 cm^−1^ is in line with the values observed for the hydroperoxides of the N6-pentamer. The major spin density is on the C-atom while still significant contributions are present on the HO_2_-part, the α-amine and the hydroperoxide group. The latter shows a small contribution to the spin density of its terminal OH-moiety. The O-O bond of the hydroperoxide is 1.457 Å, very close to its starting value of 1.451 Å. The O-O bond starts to elongate only after almost complete transfer of the α-H to HO_2_(d) In the product complex (c), the distance of the former C-H bond is further elongated to 2.348 Å, and the O-O bond of the hydroperoxide is elongated to 1.826 Å. There is no longer any spin density on H_2_O_2_, but there is still considerable spin density on the a-C-atom, and now, the major spin density is on the terminal OH-moiety of the hydroperoxide. In conclusion, the order is α-H atom abstraction, followed by a late release of OH(d).

The order for decomposition of the α-amino hydroperoxides with HO_2_(d) only partly reflects the order for H-abstraction from α-CH_2_ groups of simple primary, secondary, and tertiary amines [16], which sets a limit on the use of small model systems. For secondary amines (entries 2,4), the order is dominated by the steric hindrance between the chains of the starting α-amino hydroperoxides of the N,N-3,4-dimethyl N6 pentamer. This leads to lower activation barriers of 93.9 and 85.5 kJ/mol for entries 2 and 4, respectively. The decomposition of α-amino hydroperoxides leads to the formation of the corresponding amides and the HO(d) radical. The latter is usually converted to H_2_O via a consecutive H-atom abstraction from either HO_2_(d), H_2_O_2_, or a CH_2_-group inside the N,N-3,4-dimethyl N6 pentamer and contributes to propagation, too. The amide still contains an oxidizable CH_2_-group, and entries 5 and 6 show the activation barriers for a mono-amide- and a diamide-hydroperoxide with values of 90.3 and 103.9 kJ/mol, respectively. As the electron density on the remaining CH-group of the hydroperoxides decreases, the activation barrier for H-abstraction by HO_2_(d) increases but stays well below the activation barrier for initiation.

The formation of amides increases the mass of the original PEI-800 oligomer while lowering the H/(C+N) ratio in line with the experimental findings of Nezam et al. [10]. Figure 4 shows the decomposition of the N2-β-amino hydroperoxide with HO_2_(d) (entry 3) to the corresponding amide in three steps: (a) the starting complex, (b) the transition state, and (c) the product complex. All three structures show an H-bridge between HO_2_(d) and the secondary amine on the N2-position. In the transition state, the C-H distance of the N2-β-amino hydroperoxide is 1.423 Å, and the HOO-H distance is 1.141 Å. The product complex consists of the flat dialkyl amide, H_2_O_2_, and the HO(d) radical. The HO(d) radical is stabilized by two H-bridges, one to the amide-carbonyl oxygen and one to H_2_O_2_. This will also lead to the formation of HO_2_(d) and H_2_O, as described above, because H_2_O_2_ and HO(d) are in close vicinity, thus limiting the role of HO(d) in the propagation as chain carrying radical. Furthermore, in the case of the amide-hydroperoxides, HO_2_(d) preferably shows H-bridges to the carbonyl of the amides.

Finally, formation of the amide is strongly exothermic, with an estimate for ΔH of −180.2 kJ/mol, in line with the results of the DSC experiments of Nezam et al. [10] and the NMR results of Ahmadalinezhad et al. [13] identifying R_2_NCH_2_C=O-NR_2_ as a structural element in the air oxidation of PEI.

Decomposition of α-amino hydroperoxides by the HO(d) radical, shown in entries 7–9, leads to the formation of amides, too, H_2_O and the HO(d) radical. The activation barriers are much lower than in the case of HO_2_(d) and range from 9.3 to 21.2 kJ/mol only.

#### 3.3.2. Propagation with α- and β-Amino Peroxy Radicals

**Figure 5:**
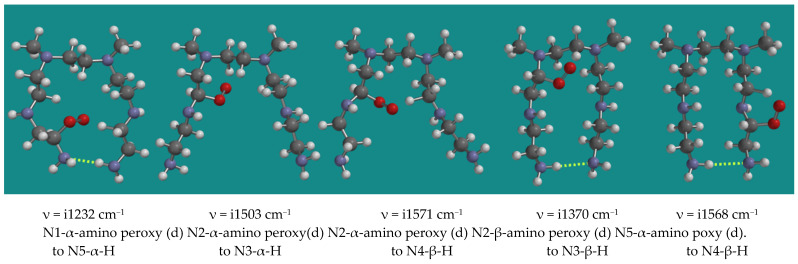
Propagation with α-amino peroxy radicals of the N,N-3,4-dimethyl N6 pentamer; B3LYP/6-31G*. Display: ball and spoke, C: grey, N: blue, H: white, hydrogen bridges: yellow.

Peroxy radicals contribute to the propagation chain. Figure 5 shows an overview of 5 transition states of the reaction of α- and β-amino peroxy radicals inside the PEI-800 model, the N,N-3,4-dimethyl N6 pentamer, as listed in Table 1, entries 10–14.

The results show a clear division between entries 10, 13, and 14 and entries 11 and 12. Entries 10, 13, and 14 show an activation barrier for internal H-abstraction of 71.3, 71.2, and 80.2 kJ/mol, respectively, while entries 11 and 12 show activation barriers of 32.4 and 36.4 kJ/mol only. The latter is due to the steric hindrance in the starting structures and the corresponding higher energy of these peroxy radicals of ~40 kJ/mol. The activation barriers are generally lower than the activation barriers for the decomposition of the α-amino hydroperoxides by the HO_2_(d) radical. These propagation reactions intrinsically are limited to occur within one PEI-800 oligomer.

#### 3.3.3. Propagation with HO_2_(d)

The propagation reaction of various N,N-3,4-dimethyl N6 pentamers and their analogues amides with HO_2_(d) were investigated, too. This is important, as the propagation reactions investigated thus far almost exclusively take place in a single PEI-800 oligomer. HO_2_(d) radical is mobile, stable, and reactive enough to be able to diffuse from one PEI-800 oligomer to another and abstract H-atoms from a variety of sources. Entries 16–19 list the results of the reactions of N,N-3,4-dimethyl N6 pentamers themselves with HO_2_(d). The activation barriers range from 84.1 for H-abstraction from N1-β-H to 107.0 kJ/mol for H-abstraction from N2-β-H. The relatively high activation barrier of this case is again due to steric hindrance around the tertiary N3 in the transition state. All starting complexes show total energies ≤ 2 kJ/mol different from each other. In all cases, the HO_2_(d) radical shows a strong H-bridge to either a secondary or a tertiary N-atom in the N,N-3,4-dimethyl N6 pentamer, very similar to the one shown and discussed in Figure 4 (a) starting complex.

**Figure 6:**
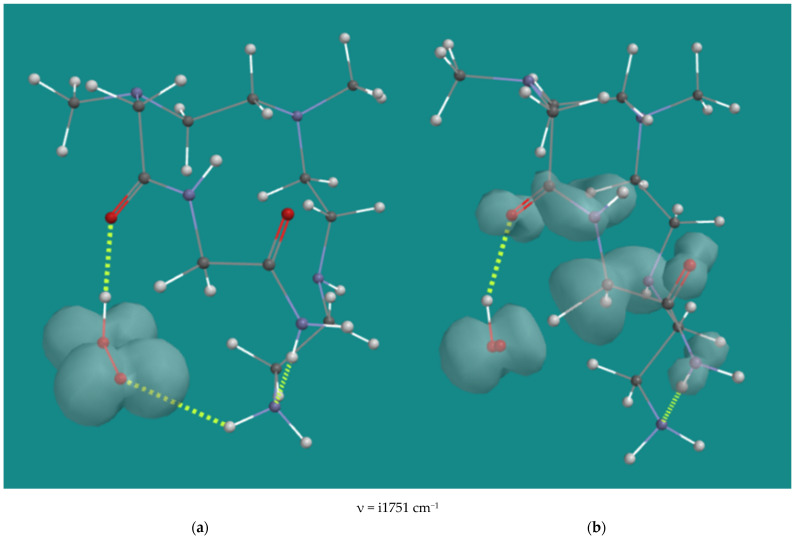
H-abstraction by HO_2_•(d) from N1,N2-diamide of N,N-3,4-dimethyl N6 pentamer; B3LYP/6-31G*. (**a**) starting complex, (**b**) transition state; Display: ball and wire, C: grey, N: blue, H: white, hydrogen bridges: yellow; surface: spin density at isosurface = 0.002 e/au^3^.

Entries 19–21 list the results of the reactions of various mono-amides derived from the original N,N-3,4-dimethyl N6 pentamer with HO_2_(d), and finally, entry 22 shows the result for the corresponding N1,N2 di-amide derived from the original N,N-3,4-dimethyl N6 pentamer. Amides are not only the principal products of the radical decomposition of α-amino hydroperoxides but should also be considered as substrates, as they still contain oxidizable CH_2_-groups. Figure 6 shows a typical example with the starting structure and the transition state of entry 22, that is, H-abstraction by HO_2_(d) from the remaining CH_2_-group of the N1,N2-diamide of the N,N-3,4-dimethyl N6 pentamer.

The starting structure shows an H-bridge of HO_2_(d) to the amide carbonyl oxygen. The OOH-O=C distance is 1.686 Å. There are also weak H-bridges between the primary amine and the oxygen of HO2(d) with a distance of 2.433 Å and between the N1 amide-NH_2_ and the N6 primary amine with a distance of 1.993 Å. In the transition state, the H-bridges of HO2(d) to the amide carbonyl oxygen and the H-bridge between the N1 amide-NH_2_ and the N6 primary amine, are maintained with distances of 1.882 Å and 1.997 Å, respectively. The C-H distance of the CH_2_-group between the two amides is 1.360 Å, and the OO-H distance is 1.195 Å. The spin density is extended to both amide groups, indicating considerable stabilization. The activation barrier for the diamide substrate is 85.8 kJ/mol, which is in the range of 70.6–100.0 kJ/mol for all amide cases listed. The H-bridge of HO_2_(d) to an amide carbonyl oxygen is a common feature of all amide starting complexes, as well as the extended spin density on the amide(s). As in the case of H-abstraction from N2-β-H by HO_2_(d), the relatively high activation barrier in this case is due to steric hindrance around the tertiary N3 in the transition state.

In short, HO_2_(d), HO(d), and α- and β-amino peroxy radicals contribute to propagation in the FCRA of PEI. All activation barriers for propagation are significantly lower than the experimental and computational activation barriers for initiation of FCRA of BPEI, which are 135.0 and 133.2 kJ/mol, respectively.

Decomposition of α-amino hydroperoxides and H-abstraction from CH_2_-groups of the N,N-3,4-dimethyl N6 pentamer by HO_2_(d) show the highest activation barriers, ranging from 84.1 to 115.7 kJ/mol. This computational range coincides very well with the range between 90 and 110 kJ/mol during propagation experimentally observed by Nezam et al. [10].

H-abstraction from CH_2_-groups of the N,N-3,4-dimethyl N6 pentamer amides by HO_2_(d) shows activation barriers, ranging from 70.6 to100.0 kJ/mol and will particularly play a role in a later stage of the oxidation with conversion ≥ 70%.

Propagation by HO(d) shows much lower activation barriers, ranging from 9.3 to 21.2 kJ/mol only, while propagation by various amino peroxy radicals shows activation barriers ranging from 32.4 to 80.2 kJ/mol. Both processes contribute to propagation but are mainly limited to a single PEI oligomer.

With some caution it can be concluded also that the exact geometry of the PEI oligomer, as has become clear from the PEI model, N,N-3,4-dimethyl N6 pentamer, can play an important role in propagation reactions, either by stabilizing or destabilizing starting structures and transition states, thus affecting activation barriers. Caution is needed because of the inherent limitations of the PEI model used and the notion that PEI oligomers are extremely flexible with a huge number of conformers.

#### 3.3.4. Propagation with Various α-Amino CH•(d) Radicals and H_2_O_2_

Thus far, propagation reactions have been considered starting from the PEI model, N,N-3,4-dimethyl N6 pentamer, and a chain carrying radicals like HO_2_(d), HO(d), or α-amino peroxy radicals. The latter is the product of a barrier-free reaction of an α-amino CH(d) radical with O_2_(t). In turn, an α-amino CH(d) radical can be the primary result of an H-abstraction by an internal amino peroxy radical, HO(d) or HO_2_(d), in the FRCA propagation chain.

**Figure 7:**
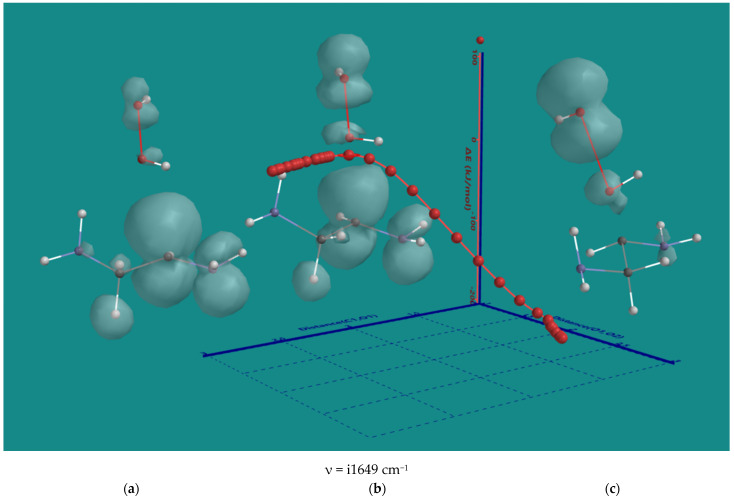
B3LYP/6-31G* IRC of the direct hydroxylation of the α-amino CH(d) radical of ethylene diamine and H_2_O_2_. (**a**) starting complex, (**b**) transition state, (**c**) product complex. Display: ball and wire, C: grey, N: blue, H: white, hydrogen bridges: yellow; surface: spin density at isosurface = 0.002 e/au^3^. ΔE is ΔE-total energy. Bond lengths in Å.

The result of H-abstraction from N,N-3,4-dimethyl N6 pentamer by HO_2_(d) is an α-amino CH(d) radical and H_2_O_2_. As H_2_O_2_ is in close vicinity to the α-amino CH(d) radical, a direct radical hydroxylation will yield an α-amino CH(OH) compound and HO(d). The nature of the direct hydroxylation was investigated using the α-amino CH(d) radical of ethylene diamine and H_2_O_2_. Figure 7 on the next page shows the results. In the starting structure (a), the spin density is mainly on the α-C. The C-O distance is 2.775 Å, and the O-O distance is 1.471 Å. Toward the transition state (b), there is a gradual decrease in the C-O distance to 2.357 Å and an increase in the O-O distance of 1.617 Å is observed. The spin density on the ethylene diamine fragment, has not changed much, but considerable spin density has developed on H_2_O_2_, particularly on the leaving OH(d). The activation barrier is ~12 kJ/mol only. The transition state can be considered as early. In the product (c), the C-O distance to 1.452 Å, and the O-O distance is 2.547 Å. There is no spin density left on the ethylene diamine fragment and the main spin density is on the OH(d) radical as the leaving group. The formation of the half-ketal can be considered as complete with a ΔE-total energy of −179 kJ/mol.

This reaction might also occur with oxidation products like the N1-amide-β-radical and the N1,N2-diamide N1-β-radical. The α-amino CH(OH) compound is a so-called half aminal. The fate of half aminals strongly depends on the reaction conditions [45]. The presence of sufficient water favors the formation of an aldehyde and an amine, while in the presence of sufficient amines, imine formation is dominant.

It should be kept in mind that the formation of an aldehyde and an amine implies chain scission in PEI. Under dry conditions, in the presence of amines and temperatures > 100 °C the half aminals might either stay partially intact or yield imine structures of the following types: RNH-CH_2_-C=N-R, RNH-C=O-C=N-R, and RNH-C=O-C=N-C=O-R. RNH-CH_2_-C=N-R and RNH-C=O-C=N-R were identified as structural units by Ahmadalinezhad and Sayari [13], but RNH-C=O-C=N-C=O-R was not.

The reaction of the α-amino CH(d) radical with H_2_O_2_ has to compete with the barrier free reaction with O_2_(t). So, an estimate was made of the relative rates of these parallel reactions, starting from the α-amino CH(d) radical, the N1-amide-β-radical, and the N1,N2-diamide N1-β-radical. As no literature data are available on neither oxygen nor H_2_O_2_ solubility in PEI-800 at temperatures between 125 °C and 150 °C, a rough estimate was made based on the ΔΔH of interaction of O_2_(t) and H_2_O_2_ and the ΔΔE_a_ of the reaction of O_2_(t) and H_2_O_2_ with an α-amino CH(d) radical of the N,N-3,4-dimethyl N6 pentamer.

Table 2 shows the results. The interaction enthalpy of O_2_ for all complexes was approximately −4.2 kJ/mol, and the activation barrier for reaction with O_2_(t) was 0.0 kJ/mol. Therefore, these values are not listed in Table 2. Furthermore, the effect of temperature turned out to be relatively small (5–25%) and not a discriminating factor between the two parallel reactions. Therefore, only the results of calculations at 137.5 °C are listed.

From Table 2 for the N1-α-amino radical and the N1-amide-β-radical, the overall reaction rate of direct hydroxylation with H_2_O_2_ is 18–19% of the rate of hydroperoxide formation. It should be kept in mind that, apart from the possible error in the calculations, the quantitative result of such a comparison is quite sensitive to the actual levels of O_2_(t) and H_2_O_2_. It is also clear that the absolute value of the H_2_O_2_ interaction enthalpy should be approximately equal to the H_2_O_2_ activation barrier, as only under these circumstances does the product of [H_2_O_2_]/[O_2_] × k-H_2_O_2_/k-O_2_ yield an r-H_2_O_2_/r-O_2_ with a considerable fraction of the overall parallel reaction rate from the hydroxylation by H_2_O_2_.

The results can be summarized as follows: air oxidation of PEI-800 leads to the formation of α-amino radicals via H-atom abstraction by O_2_(t) or HO_2_(d). In the next oxidation step, these α-amino radicals can undergo two parallel reactions:

(1) with another O_2_(t) to yield α-amino peroxy radicals;

(2) with H_2_O_2_ formed from HO_2_(d) to yield the corresponding half aminals.

Computational results of these parallel oxidation reactions indicate that reaction with H_2_O_2_ contributes considerably to propagation in PEI-800 itself, as mimicked by the N1-α-amino radical of the N,N-3,4-dimethyl N6 pentamer and its partially oxidized amide analogs. The reaction seems highly unlikely for the analogs where the amide groups are next to each other. The products of these two reactions, half aminals with structural units RNH-CH_2_-C(OH)NN-R and RNH-C=O-C(OH)NH-R, will partly lose H_2_O under dry conditions at temperatures > 125 °C to yield RNH-CH_2_-C=N-R and RNH-C=O-C=N-R structural units, which were experimentally observed by Ahmadalinezhad and Sayari [13] using advanced NMR techniques.

### 3.4. Propagation Side Reactions

#### 3.4.1. Propagation Side Reactions: β-Elimination of N-β-CH(d)NHR Radicals

While investigating propagation with α-amino peroxy radicals, a consecutive reaction was identified. The product of Table 1 entry 12, the N2-β-amino hydroperoxide-N3-β-CH•NHR(d) radical, can undergo a β-elimination leading to cleavage of C-N3 bond of the tertiary amine to yield two fragments consisting of a secondary amine radical, the N-methyl-N(d)-ethylene diamine dimer, and the N-methyl, vinyl-ethylene diamine dimer. The activation barrier is 88.3 kJ/mol, close to the value reported of 90 kJ/mol by Racicot et al. [11] for a similar reaction leading to NH_3_. This reaction could occur with a lack of oxygen. The experimental results of Nezam et al. [10] provide a clue for a lack of oxygen in the case of oxidation with 5% O_2_, which shows a weight loss of 17% compared with 12% in the case of oxidation with 17 or 30% O_2_.

**Figure 8:**
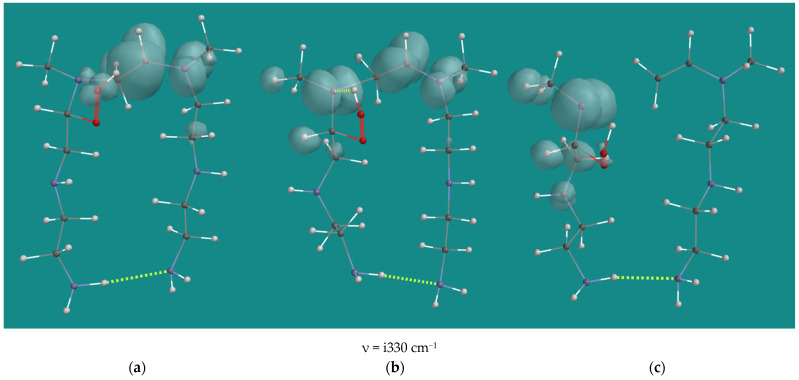
B3LYP/6-31G* β-elimination of N2-β-amino hydroperoxide-N3-β-CH•NHR(d) radical of N,N-3,4-dimethyl N6 pentamer; (**a**) starting complex, (**b**) transition state, (**c**) product complex. Display: ball and wire, C: grey, N: blue, H: white, hydrogen bridges: yellow; surface: spin density at isosurface = 0.002 e/au^3^.

Figure 8 shows the starting structure, the transition state and the product. In the starting structure, the spin density is mainly on the N3-β-carbon and N4. This is reflected in the distance between these atoms of 1.394 Å. The distance between N3-α-C and N3-β-C is 1.511 Å. The OH-group of the hydroperoxide and the N3 provide some additional stabilization to the radical. There is no clear N3-HO H-bridge, as can be seen from the distance N3-HOOR of 2.541 Å.

In the transition state, there is still a large spin density on the N3-β-carbon and N4, and the distance between these atoms of 1.368 Å has only changed slightly. The distance between N3-α-C and N3-β-C has shortened to 1.386 Å, a clear sign of the formation of a double bond. There is also a large spin density on the N3 atom, and now there is a clear N3-HO H-bridge with an N3-HOOR distance of 1.910 Å. Apparently, a radical on C or N is seriously stabilized by an OH-group in close vicinity, as this is visible in the starting structure and the transition state. The largest change is observed in the distance between N3-α-C and N3-β-C, which has enlarged to 2.141 Å, a clear sign of bond breaking.

In the product complex, the spin density on the (former) N3-β-carbon and N4 disappeared, and the formation of the double bond was completed. The spin density is now dominant on the N3-atom, with residual spin density on the α-C hydrogen atoms. The distance between N3-α-C and (former) N3-β-C was enlarged to 3.767 Å. The reaction thus far is endothermic, with ~40 kJ/mol. However, the N(d)-radical is a reactive intermediate, which will abstract an H-atom. The most favorable reaction is H-atom abstraction from the H-bridged hydroperoxide to yield the a-amino peroxy radical. This reaction has an activation barrier of 48.5 kJ/mol and an estimated reaction energy of −58.5 kJ/mol. So, the overall reaction enthalpy turns from endothermic to slightly exothermic, with −19 kJ/mol. The resulting α-amino peroxy radical will further contribute to the propagation. The visually attractive 1,2-H-shift from C to N, yielding the amide and the HO(d) radical, has an activation barrier of 164.8 kJ/mol, and it is extremely unlikely to occur.

#### 3.4.2. Propagation Side Reactions: Reactions of Alkoxy Radicals

Peroxy radicals can also undergo several self-reactions, as described in Reactions (3) and (4). These reactions have been investigated computationally in depth [42,43]. The self-reactions lead to either two alkoxy radicals and O_2_(t) (propagation Reaction (3)), a carbonyl, an alcohol compound, and O_2_(t) (termination Reaction (4)), or a peroxide and O_2_(t). These reactions presume either two RO_2_(d) radicals in close vicinity or the formation of a reactive tetroxide intermediate RO_4_R. The formation of an intermediate tetroxide turned out to be an equilibrium reaction with a ΔG ~0.0 kJ/mol for R = 2-butyl.

Assuming that the steric demands in the PEI-800 case for two peroxy radicals will be at least similar to the one observed for a single peroxy radical of ~40 kJ/mol, the self-reaction of two α-amino peroxy radicals inside the PEI-800 model seems not very plausible, neither as propagation nor as termination reaction. However, as described by Salo et al. [30], the reaction between a peroxy radical and an HO_2_(d) radical is very possible. The main product of that reaction is the corresponding hydroperoxide and O_2_(t) [15,30], but the formation of a small amount of an alkoxy radical, O_2_(t), and HO(d) is also possible, as the overall reaction energy is slightly exothermic, by −8 kJ/mol, and the reaction barriers are very low with values ranging from 0–12 kJ/mol. Alkoxy radicals are very reactive and show H-abstraction and neighboring C-C bond cleavage reactions [27]. Thus, though not of major quantitative importance for propagation, H-abstraction and C-C bond cleavage reactions of N1-, N2-, and N3-α-amino oxy radicals of the N,N-3,4-dimethyl N6 pentamer were investigated, as they might offer an explanation for some side-products. Table 3 lists an overview of the results.

**Figure 9:**
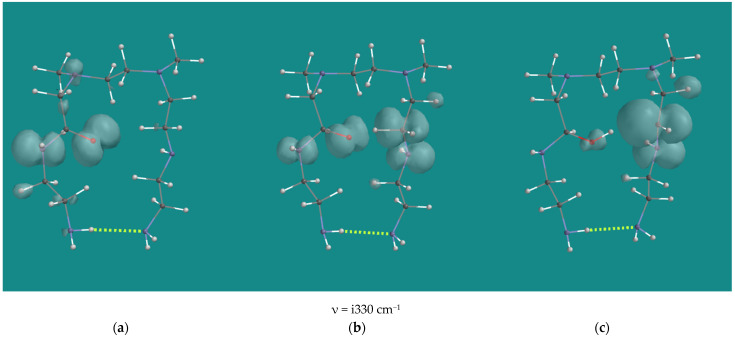
N4 β-H-abstraction of the N2-α-amino oxy radical of N,N-3,4-dimethyl N6 pentamer; B3LYP/6-31G*. (**a**) starting complex, (**b**) transition state, (**c**) product complex. Display upper: space-filling; display lower: ball and wire, C: grey, N: blue, H: white, hydrogen bridges: yellow; surface: spin density at isosurface = 0.002 e/au^3^.

Activation barriers of the C-C bond cleavage reactions are not listed, as no transition states could be located. An Energy Profile, starting from α-amino oxy radicals and increasing the C-C distance to 2.5 Å in steps of 0.1 Å, resulted in a continuous descending total energy curve as a function of the increasing distance. With some caution, it can be concluded that the C-C bond cleavage reactions are barrier free. The activation barriers for H-abstraction for the N1-α-amino oxy radical and the N2-β-amino oxy radical are close to 0 kJ/mol. With respect to the negative activation barrier for H-abstraction of the N1-α-amino oxy radical, it should be noted that the B3LYP/6-31G* total energy of the transition state is 7 kJ/mol higher than the total energy of the starting structure, but its zero-point energy is 9 kJ/mol lower, resulting in an activation barrier of −1.9 kJ/mol. The activation barrier for H-abstraction for the N2-α-amino oxy radical with 25.3 kJ/mol is significantly higher than the N1 and N2 cases. This is due to steric hindrance in the transition state, albeit not as much as in the case of the H-abstraction of the N2-α-amino peroxy (d) radical (Table 1, entry 11). Figure 9 shows an overview of the H-abstraction reaction for the N2-α-amino oxy radical. In the product, the steric hindrance still accounts for approximately 14–17 kJ/mol, as can be deducted from the difference from the ΔH H-abstraction by the N2-α-amino oxy radical compared with the ΔH H-abstraction by the N2-α-amino oxy radical and the N2-β-amino oxy radical.

Primary products of these H-abstraction reactions are again half aminals and a-amino CH(d) radicals. The radicals in turn will react with oxygen or H_2_O_2_ as discussed before and could deliver a limited contribution to propagation.

#### 3.4.3. Formation of NH_3_

In the case of a half aminal originating from a primary amine, one of the products will be NH_3_. This reaction thus provides an explanation for the formation of NH_3_ from primary amines in PEI-800.

According to Hermans et al. [27], in the FRCA of cyclohexane, the alkoxy radical yields ~60% cyclohexanol and ~40% of ω-formyl product, based on the minor differences in activation barriers between the two reactions. In the PEI-800 case, the activation barriers of both reactions are ~0 kJ/mol, resulting in a 50% contribution for each of them. Thus, the maximum amount of NH_3_ formation from initial primary amines should be ~20% because the number of primary amines in PEI-800 is ~40%, as described below Figure 1. Racicot et al. [11] reported an NH_3_ production of 21% (mmol NH_3_/mmol PEI-800). So, the computational results coincide well with the experimental ones. Finally, if NH_3_ is produced from a half aminal, the other product should be an aldehyde. However, in an environment with a high number of amines, the aldehyde will be converted rapidly to an imine and H_2_O.

#### 3.4.4. Formation of CO_2_

Products of the C-C bond cleavage reactions of α-amino oxy radicals of the N,N-3,4-dimethyl N6 pentamer are N-formamides and R_1_R_2_NCH_2_(d) radicals. The R_1_R_2_NCH_2_(d) radicals will react barrier free with oxygen to yield The R_1_R_2_NCH_2_O_2_(d) peroxy radicals, which eventually will also yield N-formamides via the corresponding hydroperoxides, as discussed earlier. Air oxidation of various formamides with the HO(d) radical as an initiator was studied experimentally and computationally by Bunkan et al. [46] in their research on atmospheric chemistry. In the gas phase, H-abstraction by the HO(d) radical from the aldehyde of N-methyl formamide is the main reaction with a yield of 83%. Related to atmospheric chemistry also, a computational study of the reaction of the HO_2_(d) radical with acetaldehyde [47] established that the HO_2_(d) radical reacts easily with acetaldehyde to yield the corresponding α-hydroxyethyl peroxy radical. The authors pointed out that the HO_2_(d) radical is present in a much higher concentration than the HO(d) radical. Therefore, in FRCA of PEI, it seems more obvious to study the reaction of formamide with HO_2_(d) than the reaction between formamide and HO(d). Formamide and N-methyl formamide were taken as small model systems for PEI. As all computational results obtained were similar for both systems, the results of formamide and N-methyl formamide will be discussed simultaneously.

Direct H-abstraction from formamide and N-methyl formamide by HO_2_(d) yielded the N-acyl radical and H_2_O_2_ with an activation barrier of 97.2 and 94.2 kJ/mol, respectively. A visually attractive consecutive reaction would be the direct formation of the carbamic acid and HO(d). However, the initial H-abstraction by HO_2_(d) was endothermic by 84.9 and 83.5 kJ/mol, respectively, and upon the desired approach of H_2_O_2_ to the N-acyl radical, the reverse reaction showed activation barriers of 12.3 and 10.7 kJ/mol only. Furthermore, no transition state for the formation of the carbamic acid and HO(d) could be determined.

**Figure 10:**
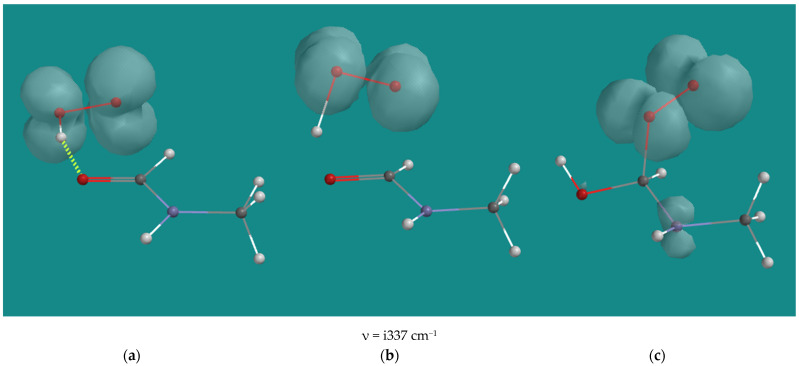
Addition of HO_2_•(d) to N-methyl formamide; B3LYP/6-31G*. (**a**) starting complex, (**b**) transition state, (**c**) product complex. Display: ball and wire, C: grey, N: blue, H: white, hydrogen bridges: yellow; surface: spin density at isosurface = 0.002 e/au^3^.

By creating an Energy Profile of the approach of HO_2_(d) to formamide or N-methyl formamide, an energy maximum with a C-O distance of ~2.0 Å and an OOH-O=C distance of ~1.4 Å was observed, indicating an HO_2_(d) addition to the carbonyl function. Transition States could be established in both cases with activation barriers of 50.5 and 51.5 kJ/mol for formamide and N-methyl formamide, respectively. Figure 10 shows the starting structure, transition state, and product structure for N-methyl formamide.

In the transition state, the OH distance was 1.087 Å, and the O-C distance was 2.024 Å, indicative of the process where proton transfer precedes O-C bond formation. The radical character remains on the O-O part, as can be seen from the spin densities in all three structures.

In the second step, H-transfer takes place with another HO_2_(d), yielding the hydroperoxide and O_2_(t). This is the usual type of low-barrier H-atom transfer, with an activation barrier of 10.2 and 8.4 kJ/mol for formamide and N-methyl formamide, respectively.

The third step is CH H-abstraction from the hydroperoxide by HO_2_(d), yielding the carbamic acid derivatives of formamide and N-methyl formamide, H_2_O_2_, and the HO(d) radical, with activation barriers of 57.2 and 59.8 kJ/mol, respectively. The HO(d) thus formed reacts with H_2_O_2_ to yield H_2_O and HO_2_(d). The activation barrier was 4.6 kJ/mol only. The carbamic acids might lose CO_2_ easily via amine catalysis.

Keeping in mind that the reaction between an N-α-amino peroxy radical of N,N-3,4-dimethyl N6 pentamer and an HO_2_(d) radical, yielding an N-α-amino oxy radical of N,N-3,4-dimethyl N6 pentamer is a side-reaction, the overall sequence of reactions provides a plausible explanation for the formation of CO_2_, consistently using HO_2_(d) as the chain carrying radical.

## 4. Discussion

### 4.1. Set of Propagation Reactions

The oxidation of PEI is described best as a BAS process or FRCA [25,26] with Initiation, Propagation, and Termination as important conceptual steps. Decomposition of the initially formed α-amino hydroperoxide is the first step in Propagation. Propagation occurs partly via α-amino CH(d) radicals, peroxy radicals, OH-radicals inside a PEI oligomer and mainly via HO_2_ radicals both inside and between different PEI oligomers.

All computational findings are supported by earlier experimental findings with respect to activation barriers, structural elements identified in the oxidized PEI, and volatile products like NH_3_, H_2_O, and CO_2_. Additional support for various steps was found in the literature on FRCA processes, like the oxidation of cyclohexane and toluene.

A specific role in the propagation plays the reaction of various α-amino CH(d) radicals of PEI with H_2_O_2_. Direct hydroxylation with H_2_O_2_ accounts for ~16% of the overall propagation. The product of that reaction is a half aminal RNH-C(OH)CH_2_NR_2_, which, in the case of a primary amine under dry conditions and T > 125 °C, leads to the formation of NH_3_ and an imine and, in the case of a secondary or tertiary amine, to chain scission and imine formation.

The computational result for NH_3_ formation quantitatively coincides with the experimental one of approximately 20%. In addition, it provides an explanation for the formation of CO_2_ via the oxidation of N-formamide species with HO_2_(d). The N-formamide species are the product of the direct hydroxylation with H_2_O_2_ or the H-abstraction reaction of alkoxy radicals, which, in both cases, eventually leads to half aminals.

Thus, a simplified overall scheme for propagation in FRCA of PEI can be set up:

PEI-α-amino CH-OOH + HO_2_(d) → PEI-amide + HO_2_(d) (12)

PEI + HO_2_•(d)      → PEI-α-amino CH(d) + H_2_O_2_ (13)

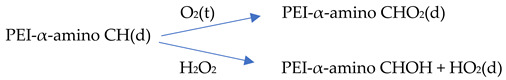
(14)

PEI-α-amino CHO_2_(d) + PEI → PEI-α-amino CH-OOH + PEI-α-amino CH(d) (15)

In this simplified scheme with five reactions only, it is important to note that is assumed that HO(d), in most cases, will react barrier free and very fast with either H_2_O_2_ or HO_2_(d) to yield H_2_O and HO_2_(d) or O_2_(t) because H_2_O_2_ and HO_2_(d) are present in close vicinity. Additionally, H-abstraction from PEI by HO(d) could occur to yield H_2_O and PEI-α-amino CH(d).

The activation barriers for the H-abstraction from PEI-α-amino CH-OOH and PEI itself by HO_2_(d) range from 84.1 to 115.7 kJ/mol or approximately 100 kJ/mol on average, in line with the experimental findings from Nezam et al. [10] of 105 ± 10 kJ/mol for the overall activation barrier of FRCA of PEI up to 60% conversion and in line with the observation that loss of amine efficiency precedes heat production.

The reaction of PEI-α-amino CH(d) with O_2_(t) is barrier free. The reaction of PEI-α-amino CH(d) with H_2_O_2_ shows an activation barrier of 37.1 kJ/mol, while the analog reaction of an N-amide-β-radical shows an activation barrier of 60.8 kJ/mol. Finally, the reaction of PEI-α-amino CHO_2_•(d) with PEI shows activation barriers ranges from 32.4 to 80.2 kJ/mol.

It can be concluded that up to a conversion of approximately 60%, FCRA of PEI is dominated by H-abstraction from PEI-α-amino CH-OOH by HO_2_(d) and that, thereafter, H-abstraction from PEI and PEI-amides by HO_2_(d) takes over with slightly lower average activation barriers.

### 4.2. Final Products of FRCA of PEI

Thus far, the focus has been entirely on reactions contributing to the propagation in FCRA of PEI. Experimentally determined volatile products like NH_3_, H_2_O, and CO_2_ and elucidated structural elements in the final product like amides and several types of imines were used to establish the plausibility of the reactions computationally investigated. In this section, an attempt will be made to describe the final product of FRCA of PEI, as described by Nezam et al. [10]. Fully oxidized PEI-800 still contains 3.5 H per repeat unit compared with 5 H per repeat unit in pristine PEI, and the chain length of PEI has dropped from 19 to 15. The mass retained after complete oxidation is approximately 87% as a net result of mass increase due to the incorporation of oxygen and mass loss due to the production of NH_3_, H_2_O, and CO_2_. The amount of NH_3_ produced was 0.2 mol/mol repeat units in PEI, corresponding to approximately 50% of the primary amines in pristine PEI and the corresponding C/N ratio. The amount of H_2_O produced was 0.5 mol/mol repeat unit in PEI, while the apparent CO_2_ production is very low with ~0.01 mol/mol repeat unit [10,11]. All experimental data were combined and put in Table 4 to obtain an idea of what the fully oxidized PEI oligomer looks like and what the contribution of the different reactions identified is to the final product.

The left side of Table 4 contains input data. It starts with trivial names for structural PEI repeat units, which are specified in the next column with a formula description. The third column contains the molecular weight of these units. The fourth and fifth columns contain their corresponding absolute H/(C+N) and N/C ratios. The N/C ratio is the inverse of the C/N ratio, as reported by Racicot et al. [11]. This is done to avoid division by zero in the case of the entry imine-NH_3_, representing the case of NH_3_ loss from primary amines. The middle column (grey background) contains the number (n) of the various structural PEI repeat units contributing to the fully oxidized PEI-800 oligomer. Varying these numbers leads to different results. The basic condition is that it should sum up to 15, as experimentally obtained [10]. The columns to the right side of the table contain resulting calculated ratios c-H/(C+N), c-N/C, and c-Mw for all structural PEI repeat units. The last two rows, Sum and Experimental values (Exp. Values) contain the sum of the various structural PEI repeat units (n), the weighted sum of the calculated ratios c-H/(C+N), c-N/C., and the calculated molecular weight of the fully oxidized PEI-800 oligomer. The experimental values for H/(C+N) and N/C vary slightly with temperature and oxygen concentration.

The molecular weight of the pristine PEI-800 oligomer is 834 g/mol, as shown in and described below in Figure 1. According to Nezam et al. [10], 83% of the mass is retained in fully oxidized PEI-800 at 5% O_2_ and 87% at 17 and 30% O_2_. The latter value was taken as a reference, and thus, the mass of a fully oxidized PEI-800 oligomer is 834 g/mol*0.87 = 725.6 g/mol, listed in the row Experimental value. From the comparison of the calculated values with the experimentally observed values in the last three columns, it can be seen that they agree reasonably well.

The purpose of Table 4 was not to obtain a perfect fit with all experimental data but to obtain a general impression of the composition of the fully oxidized PEI-800 and compare that to the computational values of the reactions described, leading to the various structural PEI repeat units. Therefore, the resolution of n was limited 0.5 units.

Surprisingly, not all CH_2_ groups are oxidized in the fully oxidized PEI-800 oligomer, and probably even more surprising is the fact that not even one single CH_2_ group in all repeat units is oxidized. Pristine PEI contains 5 H atoms per repeat unit. Fully oxidized PEI still contains ~3.5 H atoms per repeat unit. A direct consequence of this experimental finding is that the FRCA of PEI-800 does not lead to 100% amide repeat units, as the amide still contains 3.0 H per repeat unit. From Table 4, it becomes plausible that even pristine PEI repeat units should be present in order to arrive at the overall composition in line with all experimental values. Pristine PEI and half aminal PEI repeat units both contain 5 H atoms per unit, but increasing the contribution of the half aminal at the cost of pristine PEI increases the molecular weight substantially to 746 g/mol, corresponding to 90% mass retained, which is not in line with the experimental findings. So, it seems that ~10% of pristine PEI repeat units are not oxidized under these conditions. A plausible explanation could be the formation of ammonium bicarbonates, which are more difficult to oxidize [15], and steric hindrance inside PEI-800 oligomers, which prevents propagation by HO_2_(d) for specific geometries.

The total amount of α-amino hydroperoxide decomposition leads to ~ 47% of amide products, built up from six amide repeat units and one amide-imine unit on a total of 15 repeat units. The total amount of direct hydroxylation of α-amino PEI radicals by H_2_O_2_ in the propagation accounts for ~60%, built up from 3.5 half aminals units, 1.5 imine units, 3.0 imine-NH_3_ units, and 1.0 imine-amide units on a total of 15 repeat units.

At first glance, this seems significantly larger than the estimated 16%, which is calculated under Section 3.3.4. Propagation with various α-amino CH(d) radicals and H_2_O_2_. However, with an overall error of 5 kJ/mol, a contribution of ~80% to propagation is within the error limit. This does not disqualify the computational results obtained but gives an impression of the sensitivity of the systems. The loss of 3.0 imine-NH_3_ units corresponds to a ~16% loss of all amine groups, more precisely NH_3_, in line with the experimental findings and the computational result of ~20%. However, the experimentally observed CO_2_ production is too low to account for the remaining amount of mass loss. The same is true for the experimentally observed loss of H_2_O. A plausible explanation could be that they are stored as various secondary and tertiary ammonium bicarbonates, as described above.

There is a rather small variation in the retained mass on full oxidation of PEI-800 as a function of the oxygen concentration. At 5% of O_2_, the retained mass is ~83%, while at 17 and 30% of O_2_, the retained mass is ~87%. An obvious way to explain the difference between these values is that at 5% O_2_, less oxygen is incorporated than at 17 and 30% O_2_. This might be considered as a sign that some oxygen mass transfer limitation occurs at 5%; however, this seems not to be the case with 17 and 30% O_2_. With some caution, it can be concluded that oxygen mass transfer limitation was not dominant at the experimental conditions applied, which in itself is in line with a reaction order for O_2_ of ~0.5–0.7 [10] and the analysis of Hoorn et al. [33] regarding toluene oxidation under industrial conditions at similar temperatures that FRCA of toluene is a slow chemical reaction compared to physical mass transfer of oxygen.

Most of the experimental and computational work applies to BPEI; however, the experimental work of Ahmadalinezhad and Sayari [13] provides information on LPEI too. The major difference was the absence of imine-amide PEI repeat units, which is easily explained by the almost absence of primary amines in LPEI compared with BPEI.

## 5. Conclusions

In this article, a computational study was conducted to Propagation in FRCA of (B)PEI. This study was calibrated using experimental data on air oxidation of BPEI itself and well-known large scale industrial oxidation processes like cyclohexane and toluene oxidation. In addition, the literature results of computational studies on cyclohexane and toluene oxidation were also used.

From the computational on the propagation in FRCA of PEI, the following can be concluded:

1. The initial formation of α-amino hydroperoxides provides a good explanation for the experimental finding that loss of CO_2_ efficiency precedes major heat production in the FCRA of PEI;

2. α-H atom abstraction from α-amino hydroperoxides of PEI by HO_2_(d) is a crucial step in the propagation, which yields corresponding amide PEI repeat units, H_2_O_2_, and OH(d);

3. The very reactive OH(d) reacts almost barrier free with H_2_O_2_ and HO_2_(d) to yield HO_2_(d) and O_2_(t) respectively but still might play a limited role in propagation inside a PEI oligomer;

4. HO_2_(d) is the most important chain carrying radical in the propagation as it not only can react inside a PEI oligomer but also can transfer the radical chain to other PEI oligomers;

5. The reaction of HO_2_(d) with a PEI oligomer leads to a PEI-α-amino CH(d) radical and H_2_O_2_;

6. The PEI-α-amino CH(d) radical reacts with O_2_(t) in a barrier free process to yield the PEI-α-amino peroxy radical, which in turn abstracts an α-H-atom inside a PEI oligomer in a process to yield PEI-α-amino hydroperoxides with moderate activation barriers. Next, the PEI-α-amino hydroperoxides react with HO_2_(d) to yield the corresponding amides as described in conclusion 2. This is a well-known sequence in propagation;

7. In a parallel propagation step, the PEI-α-amino CH(d) radicals react directly with H_2_O_2_ to yield half aminals with the general structure: PEI-α-amino CH(OH);

8. The relative contribution of both reactions to propagation is about 50% for each of them, as calculated from the total amount of amide and imine derived PEI repeat elements in the fully oxidized PEI;

9. The half aminals with structure PEI primary amine α-amino CH(OH) are the main source for the formation of NH_3_, and the computational results provide a quantitative explanation for the amount of NH_3_ produced;

10. Half aminals are also the source of various imine structures, identified with advanced NMR techniques;

11. The results of elemental analysis strongly suggest that not all pristine PEI repeat units are oxidized and that approximately 10% remains unaffected. Computational results suggest the formation of ammonium bicarbonates and steric hindrance inside a PEI oligomer as possible explanations;

12. Combination of experimental and computational results lead to a semi-quantitative account of the structure of the fully oxidized PEI and the NH_3_ produced but not for the corresponding amounts of H_2_O and CO_2_. A plausible explanation for this discrepancy is the formation of non-volatile secondary and tertiary ammonium bicarbonate species.

## Figures and Tables

**Table 1:** Overview B3LYP/6-31G* activation barriers of propagation reactions with various N,N-3,4-dimethyl N6 pentamers and their α-amino hydroperoxides.

Entry	N,N-3,4-Dimethyl N6 Pentamer and R(d)		E_a_ (kJ/mol)	ν (cm^−1^)
1	N1-α-amino hydroperoxide	HO_2_(d)	115.7	i1790
2	N2-α-amino hydroperoxide	HO_2_(d)	93.9	i1486
3	N2-β-amino hydroperoxide	HO_2_(d)	112.3	i1597
4	N5-α-amino hydroperoxide	HO_2_(d)	85.5	i1265
5	N1-amide-β-hydroperoxide	HO_2_(d)	90.3	i1401
6	N1,2-diamide-N1-β-hydroperoxide	HO_2_(d)	103.9	i1639
7	N1-α-amino hydroperoxide	HO(d)	21.2	i794
8	N2-β-amino hydroperoxide	HO(d)	9.3	i989
9	N4-β-amino hydroperoxide	HO(d)	9.9	i131
10	N5-α-H	N1-α-amino peroxy radical (d)	71.3	i1232
11	N3-α-H	N2-α-amino peroxy radical (d)	32.4	i1503
12	N4-β-H	N2-α-amino peroxy radical (d)	36.2	i1571
13	N3-β-H	N2-β-amino peroxy radical (d)	71.2	i1370
14	N4-β-H	N5-α-amino peroxy radical (d)	80.2	i1568
15	N1-α-H	HO_2_(d)	92.9	i1507
16	N1-β-H	HO_2_(d)	84.3	i1375
17	N2-β-H	HO_2_(d)	107.0	i1510
18	N3-β-H	HO_2_(d)	85.1	i1524
19	N1-amide	HO_2_(d)	70.6	i1223
20	N2-amide	HO_2_(d)	100.0	i1668
21	N3-amide	HO_2_(d)	83.1	i1643
22	N1,N2-diamide	HO_2_(d)	85.8	i1751

**Table 2:** Overview B3LYP/6-31G* interaction enthalpies with H_2_O_2_, activation barriers for hydroxylation by H_2_O_2_ of various N,N-3,4-dimethyl N6 pentamer radicals, and the resulting estimates for [H_2_O_2_]/[O_2_], k-H_2_O_2_/k-O_2_, and r-H_2_O_2_/r-O_2_ at 137.5 °C. All calculations are based on the simple approximation: ΔG~ΔH = −RT*ln K and ΔE_a_~ΔH_a_ = −RT*ln k.

N,N-3,4-Dimethyl N6 Pentamer Radical	ΔH H_2_O_2_ (kJ/mol)	E_a_ H_2_O_2_ (kJ/mol)	[H_2_O_2_]/[O_2_]	k-H_2_O_2_/k-O_2_	r -H_2_O_2_/r-O_2_
N1-α-amino radical	−35.4	37.1	9.37 × 10^3^	1.90 × 10^−5^	1.78 × 10^−1^
N1-amide-β-radical	−59.3	60.8	1.04 × 10^7^	1.83 × 10^−8^	1.89 × 10^−1^
N1,N2-diamide N1-β-radical	−58.2	90.9	7.40 × 10^6^	2.70 × 10^−12^	2.00 × 10^−5^

**Table 3:** Overview B3LYP/6-31G* activation barriers and reaction enthalpy H-abstraction and reaction enthalpy C-C bond cleavage reactions of N1-, N2-, and N3-α-amino oxy radicals of N,N-3,4-dimethyl N6 pentamer.

N,N-3,4-Dimethyl N6 Pentamer	ν TS	E_a_ H-Abstraction	ΔH H-Abstraction	ΔH C-C Cleavage
	cm^−1^	kJ/mol		
N1-α-amino oxy radical	i615	−1.9	−39.2	−92.0
N2-α-amino oxy radical	i1310	25.3	−22.4	−116.2
N2-β-amino oxy radical	i325	0.9	−36.6	−113.1

**Table 4:** Composition of the various structural elements in a fully oxidized PEI-800 oligomer combined with values obtained from elemental analysis, volatile products measured, and mass retained.

Name	PEI Unit	Mw	H/(C+N)	N/C	n	c-H/(C+N)	c-N/C	c-Mw
		g/mol	-	-	-	-	-	g/mol
pristine	CH_2_CH_2_NH	43	1.67	0.50	1.5	2.50	0.75	64.5
amide	C=O-CH_2_NH	57	1.00	0.50	6.0	6.00	3.00	342.0
half aminal	CH(OH)-CH_2_NH	59	1.67	0.50	2.0	3.33	1.00	118.0
imine	CH_2_CH=N	41	1.00	0.50	1.5	1.50	0.75	61.5
imine-NH_3_	CH_2_CH	27	1.50	0.00	3.0	4.50	0.00	81.0
imine-amide	C=O-CH=N	55	0.33	0.50	1.0	0.33	0.50	55.0
Sum	-	-	-	-	15.0	1.21	0.40	722.0
Exp. values	-	-	-	-	15.0	1.25–1.50	0.42–0.43	725.6

## Data Availability

All data are contained in the article and Supplementary Materials.

## Supplementary Materials

The following supporting information can be downloaded at https://www.mdpi.com/article/10.3390/nano15040313/s1, Molecular structures (.mol2) (ZIP), Propagation (.xslx), Guidance Supplementary Materials (PDF).
