# Supplementary material for: A Molecular Modeling Study on the Propagation in Free Radical Chain Oxidation of (B)PEI
Source: Nanomaterials (Basel). 2025 Feb 18;15(4):313. doi: 10.3390/nano15040313 (PMC11858157; doi:10.3390/nano15040313)
Supplement: Supplementary file 1 [file nanomaterials-15-00313-s001.zip › Supplementary Materials/Guidance Supllementary Materials.pdf]

Guidance Supplementary Materials

A Molecular Modeling Study on the Propagation in Free Radical Chain Oxidation of (B)PEI.

Wim Buijs* orcid.org/0000-0003-3273-5063

* Correspondence: wbuijsm@gmail.com

Contents

Supplementary Materials contains:

1. Propagation (Excel; xslx).
2. All molecular structures: [name.mol2]

Ad 1: The Propagation excel file contains all relevant data of the article:

Sheet 1 Calculations: Primary molecular modelling data (total energies, enthalpy corrections, unique imaginary frequency numbers, activation barriers and reaction enthalpies. Data follow the order of first appearance in the article.

Sheet 2: Table 4. The table appears as described in the article. However, column G (blue fill color, heading n) might be adapted to see the effect on columns H, I, J with respect to the experimental values shown in row 12.

Ad 2.: The molecular structures are listed in a .zip file with folders according to the figures and tables in the manuscript for easy access. In order to restore all quantitative data from the .mol2 files, a single-point calculation and a frequency calculation should be performed using the method listed in the name: B3LYP/6-31G*. A frequency calculation is required to restore the thermodynamical enthalpy corrections, including the Zero Point Energy (ZPE).

B3LYP is applied as originally described, using for Exchange: 0.2000 Hartree-Fock + 0.0800 Slater + 0.7200 B88 and for Correlation: 0.1900 VWN1RPA + 0.8100 LYP

General Convergence Criteria:
SCF tolerance = $1*10^{-7}$ Hartree; Geometry optimization Gradient tolerance = $7*10^{-4}$ Hartree/Bohr and the Distance tolerance =$1.4*10^{-3}$ Å

All calculations can in principle be carried out with a variety of (commercial) molecular simulations packages like Spartan, GAUSSIAN, Materials Studio or SCN/AMS.
